# Supplementary material for: Modeling glioblastoma heterogeneity as a dynamic network of cell states
Source: Mol Syst Biol. 2021 Sep 16;17(9):e10105. doi: 10.15252/msb.202010105 (PMC8444284; doi:10.15252/msb.202010105)
Supplement: Supplementary file 6 — Source Data for Figure 5 [file MSB-17-e10105-s004.zip › Figure5A_sourcedata/GSEA_3071/hallmarks_stateV.GseaPreranked.1623742546329/HALLMARK_MITOTIC_SPINDLE.html]

Details for gene set HALLMARK\_MITOTIC\_SPINDLE[GSEA]

|  || Dataset | state43071 |
| Phenotype | NoPhenotypeAvailable |
| Upregulated in class | na\_pos |
| GeneSet | HALLMARK\_MITOTIC\_SPINDLE |
| Enrichment Score (ES) | 0.36474985 |
| Normalized Enrichment Score (NES) | 2.0520132 |
| Nominal p-value | 0.0 |
| FDR q-value | 6.2741374E-4 |
| FWER p-Value | 0.008 |
Table: GSEA Results Summary

  

Fig 1: Enrichment plot: HALLMARK\_MITOTIC\_SPINDLE      
 Profile of the Running ES Score & Positions of GeneSet Members on the Rank Ordered List

  

| PROBE | GENE SYMBOL | GENE\_TITLE | RANK IN GENE LIST | RANK METRIC SCORE | RUNNING ES | CORE ENRICHMENT || 1 | NUSAP1 |  |  | 6 | 2.001 | 0.0224 | Yes |
| 2 | TOP2A |  |  | 14 | 1.842 | 0.0428 | Yes |
| 3 | CENPF |  |  | 28 | 1.705 | 0.0604 | Yes |
| 4 | KIF15 |  |  | 52 | 1.568 | 0.0745 | Yes |
| 5 | CDK1 |  |  | 69 | 1.488 | 0.0889 | Yes |
| 6 | FBXO5 |  |  | 82 | 1.422 | 0.1034 | Yes |
| 7 | SMC4 |  |  | 127 | 1.320 | 0.1105 | Yes |
| 8 | LMNB1 |  |  | 165 | 1.238 | 0.1179 | Yes |
| 9 | KIF23 |  |  | 167 | 1.235 | 0.1323 | Yes |
| 10 | KIF4A |  |  | 191 | 1.193 | 0.1419 | Yes |
| 11 | PRC1 |  |  | 211 | 1.161 | 0.1519 | Yes |
| 12 | KIF22 |  |  | 213 | 1.157 | 0.1654 | Yes |
| 13 | NDC80 |  |  | 240 | 1.129 | 0.1737 | Yes |
| 14 | TPX2 |  |  | 248 | 1.122 | 0.1856 | Yes |
| 15 | SMC1A |  |  | 251 | 1.120 | 0.1984 | Yes |
| 16 | SAC3D1 |  |  | 252 | 1.118 | 0.2116 | Yes |
| 17 | BRCA2 |  |  | 274 | 1.089 | 0.2204 | Yes |
| 18 | BUB1 |  |  | 277 | 1.084 | 0.2328 | Yes |
| 19 | CDK5RAP2 |  |  | 292 | 1.076 | 0.2428 | Yes |
| 20 | ECT2 |  |  | 295 | 1.071 | 0.2550 | Yes |
| 21 | KNTC1 |  |  | 342 | 1.023 | 0.2582 | Yes |
| 22 | KIF11 |  |  | 369 | 0.982 | 0.2648 | Yes |
| 23 | TTK |  |  | 383 | 0.966 | 0.2736 | Yes |
| 24 | PCM1 |  |  | 386 | 0.964 | 0.2846 | Yes |
| 25 | DLGAP5 |  |  | 418 | 0.936 | 0.2897 | Yes |
| 26 | CEP192 |  |  | 429 | 0.924 | 0.2987 | Yes |
| 27 | CENPE |  |  | 437 | 0.918 | 0.3081 | Yes |
| 28 | INCENP |  |  | 440 | 0.916 | 0.3185 | Yes |
| 29 | RACGAP1 |  |  | 443 | 0.912 | 0.3289 | Yes |
| 30 | CENPJ |  |  | 479 | 0.885 | 0.3326 | Yes |
| 31 | PLK1 |  |  | 526 | 0.850 | 0.3337 | Yes |
| 32 | BIRC5 |  |  | 541 | 0.842 | 0.3410 | Yes |
| 33 | MID1 |  |  | 574 | 0.815 | 0.3444 | Yes |
| 34 | FGD4 |  |  | 624 | 0.787 | 0.3442 | Yes |
| 35 | PIF1 |  |  | 632 | 0.782 | 0.3521 | Yes |
| 36 | SMC3 |  |  | 708 | 0.743 | 0.3464 | Yes |
| 37 | NIN |  |  | 712 | 0.742 | 0.3546 | Yes |
| 38 | CKAP5 |  |  | 752 | 0.723 | 0.3556 | Yes |
| 39 | AURKA |  |  | 761 | 0.718 | 0.3625 | Yes |
| 40 | MYO9B |  |  | 842 | 0.682 | 0.3551 | Yes |
| 41 | PCNT |  |  | 857 | 0.675 | 0.3604 | Yes |
| 42 | NEK2 |  |  | 901 | 0.659 | 0.3598 | Yes |
| 43 | KIF20B |  |  | 934 | 0.647 | 0.3613 | Yes |
| 44 | FARP1 |  |  | 956 | 0.638 | 0.3647 | Yes |
| 45 | CNTRL |  |  | 1011 | 0.617 | 0.3616 | No |
| 46 | CEP57 |  |  | 1053 | 0.607 | 0.3609 | No |
| 47 | MYH10 |  |  | 1172 | 0.571 | 0.3448 | No |
| 48 | KIF2C |  |  | 1309 | 0.530 | 0.3248 | No |
| 49 | CCNB2 |  |  | 1373 | 0.513 | 0.3187 | No |
| 50 | DYNLL2 |  |  | 1389 | 0.509 | 0.3218 | No |
| 51 | SUN2 |  |  | 1390 | 0.508 | 0.3278 | No |
| 52 | TUBGCP3 |  |  | 1394 | 0.508 | 0.3332 | No |
| 53 | RASA2 |  |  | 1396 | 0.507 | 0.3390 | No |
| 54 | ALMS1 |  |  | 1405 | 0.503 | 0.3434 | No |
| 55 | SHROOM2 |  |  | 1431 | 0.497 | 0.3444 | No |
| 56 | PREX1 |  |  | 1439 | 0.495 | 0.3489 | No |
| 57 | PPP4R2 |  |  | 1484 | 0.486 | 0.3462 | No |
| 58 | DLG1 |  |  | 1491 | 0.482 | 0.3507 | No |
| 59 | RFC1 |  |  | 1499 | 0.480 | 0.3550 | No |
| 60 | CCDC88A |  |  | 1564 | 0.466 | 0.3482 | No |
| 61 | SASS6 |  |  | 1618 | 0.457 | 0.3433 | No |
| 62 | TBCD |  |  | 1624 | 0.454 | 0.3477 | No |
| 63 | ESPL1 |  |  | 1733 | 0.430 | 0.3319 | No |
| 64 | PLEKHG2 |  |  | 1795 | 0.419 | 0.3251 | No |
| 65 | NET1 |  |  | 1801 | 0.417 | 0.3290 | No |
| 66 | NUMA1 |  |  | 1832 | 0.413 | 0.3281 | No |
| 67 | TUBGCP5 |  |  | 1899 | 0.404 | 0.3201 | No |
| 68 | CYTH2 |  |  | 1938 | 0.398 | 0.3175 | No |
| 69 | SSH2 |  |  | 1942 | 0.398 | 0.3216 | No |
| 70 | ANLN |  |  | 2125 | 0.371 | 0.2909 | No |
| 71 | HOOK3 |  |  | 2399 | 0.335 | 0.2421 | No |
| 72 | KATNA1 |  |  | 2424 | 0.332 | 0.2414 | No |
| 73 | TAOK2 |  |  | 2487 | 0.324 | 0.2333 | No |
| 74 | APC |  |  | 2492 | 0.323 | 0.2363 | No |
| 75 | RABGAP1 |  |  | 2498 | 0.323 | 0.2391 | No |
| 76 | RANBP9 |  |  | 2545 | 0.317 | 0.2340 | No |
| 77 | NCK1 |  |  | 2553 | 0.316 | 0.2364 | No |
| 78 | RAPGEF6 |  |  | 2580 | 0.313 | 0.2350 | No |
| 79 | ARHGEF3 |  |  | 2608 | 0.309 | 0.2335 | No |
| 80 | KIF3C |  |  | 2716 | 0.298 | 0.2163 | No |
| 81 | RHOT2 |  |  | 2785 | 0.291 | 0.2066 | No |
| 82 | CNTROB |  |  | 2918 | 0.277 | 0.1844 | No |
| 83 | ABR |  |  | 2961 | 0.273 | 0.1795 | No |
| 84 | CEP72 |  |  | 2974 | 0.272 | 0.1804 | No |
| 85 | CLIP2 |  |  | 3008 | 0.268 | 0.1772 | No |
| 86 | HDAC6 |  |  | 3048 | 0.264 | 0.1728 | No |
| 87 | UXT |  |  | 3199 | -0.251 | 0.1468 | No |
| 88 | CTTN |  |  | 3201 | -0.251 | 0.1496 | No |
| 89 | ARFIP2 |  |  | 3405 | -0.283 | 0.1138 | No |
| 90 | SHROOM1 |  |  | 3441 | -0.288 | 0.1104 | No |
| 91 | CDC42EP2 |  |  | 3688 | -0.329 | 0.0668 | No |
| 92 | WASF2 |  |  | 3692 | -0.330 | 0.0701 | No |
| 93 | GSN |  |  | 3695 | -0.331 | 0.0736 | No |
| 94 | CDC42EP1 |  |  | 3797 | -0.349 | 0.0582 | No |
| 95 | NF1 |  |  | 3882 | -0.364 | 0.0463 | No |
| 96 | EPB41L2 |  |  | 3911 | -0.369 | 0.0453 | No |
| 97 | RASA1 |  |  | 3944 | -0.377 | 0.0435 | No |
| 98 | EZR |  |  | 4058 | -0.401 | 0.0265 | No |
| 99 | NEDD9 |  |  | 4164 | -0.428 | 0.0112 | No |
| 100 | BCAR1 |  |  | 4249 | -0.456 | 0.0004 | No |
| 101 | AKAP13 |  |  | 4289 | -0.470 | -0.0016 | No |
| 102 | NOTCH2 |  |  | 4355 | -0.488 | -0.0084 | No |
| 103 | FLNB |  |  | 4707 | -0.649 | -0.0685 | No |
| 104 | MID1IP1 |  |  | 4757 | -0.683 | -0.0699 | No |
| 105 | CDC42 |  |  | 4801 | -0.716 | -0.0697 | No |
| 106 | PALLD |  |  | 4839 | -0.749 | -0.0680 | No |
| 107 | PDLIM5 |  |  | 4907 | -0.820 | -0.0713 | No |
| 108 | FLNA |  |  | 4948 | -0.862 | -0.0688 | No |
| 109 | TUBA4A |  |  | 4987 | -0.909 | -0.0655 | No |
| 110 | MYH9 |  |  | 5090 | -1.125 | -0.0719 | No |
| 111 | SORBS2 |  |  | 5144 | -1.282 | -0.0670 | No |
| 112 | STK38L |  |  | 5162 | -1.381 | -0.0540 | No |
| 113 | VCL |  |  | 5222 | -1.678 | -0.0456 | No |
| 114 | BIN1 |  |  | 5273 | -2.451 | -0.0263 | No |
| 115 | ARHGAP29 |  |  | 5278 | -2.576 | 0.0033 | No |
Table: GSEA details [plain text format]

  

Fig 2: HALLMARK\_MITOTIC\_SPINDLE: Random ES distribution      
 Gene set null distribution of ES for **HALLMARK\_MITOTIC\_SPINDLE**
